# Personality functioning as a pathway from childhood trauma to substance use in clinical and community samples

**DOI:** 10.3389/fpsyg.2026.1853182

**Published:** 2026-08-05

**Authors:** Jasmin Brouschek, Elisa Renner, Raphael Wimmer, Zoe Zipper, Hannah Brössler, Jürgen Fuchshuber, Wolfgang Beiglböck, Human-Friedrich Unterrainer

**Affiliations:** 1 Faculty of Psychotherapy Science, Sigmund Freud University Vienna, Vienna, Austria; 2 Faculty of Psychology, Sigmund Freud University Vienna, Vienna, Austria; 3 Institute of Psychology, University of Graz, Graz, Austria; 4 Department of Psychoanalysis and Psychotherapy, Medical University Vienna, Vienna, Austria; 5 Comprehensive Center for Clinical Neurosciences and Mental Health, Medical University Vienna, Vienna, Austria; 6 Department of Religious Studies, University of Vienna, Vienna, Austria; 7 Addiction Research Hub, Grüner Kreis Ltd, Vienna, Austria; 8 Department of Psychiatry and Psychotherapeutic Medicine, Medical University of Graz, Graz, Austria

**Keywords:** Bayesian modelling, childhood trauma, mentalization, personality structure, structural equation modeling, substance use

## Abstract

**Background:**

Traumatic childhood experiences are associated with an increased risk of substance abuse. It remains unclear to what extent this association is mediated by personality organization and mentalization ability across different populations.

**Method:**

A structural equation model (SEM) with bootstrap estimates was calculated in a community sample (*N* = 498), and Bayesian path analysis with informed priors was calculated in an inpatient sample of individuals diagnosed with substance use disorder (SUD sample) (*N* = 68) to examine direct and indirect relationships between childhood trauma, personality organization, mentalization ability, and addictive behavior.

**Results:**

In the community sample, structural personality impairments partially mediated the effect of childhood trauma on addictive behavior (indirect: *β* = 0.14, *p* < 0.001; direct: *β* = 0.19, *p* = 0.002), whereas mentalization showed no independent effect. In the SUD sample, childhood trauma was associated with structural impairments (*β* = 0.24), which, in turn, were associated with addictive behavior (*β* = 0.22).

**Conclusion:**

These findings highlight the central role of structural personality impairments in the association between childhood trauma and addictive behavior. While structural deficits act as a partial mediator in the general population, they emerge as the strongest and most consistent correlate of addictive behavior in clinical SUD model.

## 1 Introduction

Substance use disorders (SUDs) remain one of the greatest challenges in modern healthcare and are associated with significant socioeconomic costs and profound psychological suffering for those who are affected (American Psychiatric Association, 2022; United Nations Office on Drugs and Crime [UNODC], 2025; World Health Organization, 2025). Although neurobiological and behavioral correlates of SUD have been well researched, the underlying psychological instances that can make individuals vulnerable to addictive behavior have not been fully studied. A large number of studies show that traumatic childhood experiences, such as emotional or physical neglect/abuse or sexual abuse, can be predictors for the development of SUD (Dube et al., 2003; Felitti et al., 1998; Hyman et al., 2008; Teicher and Samson, 2016). Furthermore, the results from large meta-analyses show that the risk of addiction problems later in a person’s life increases with the number of stressful childhood experiences. Individuals with higher cumulative trauma exposure may be more likely to report substance abuse later in life (Hughes et al., 2017; Norman et al., 2012). Even when social factors and genetic influences are considered, childhood trauma remains consistently associated with an increased risk of later SUD. While the association between childhood trauma and SUD has been well established in the literature, the psychological mechanisms underlying this association can be studied further.

Theoretical frameworks, such as the self-medication hypothesis (Khantzian, 1985, 1997), propose that substance use may serve as a coping strategy for overwhelming emotional states related to traumatic experiences in early childhood, particularly when these frameworks had a negative impact on personal development (Costanzo et al., 2023; Hawn et al., 2020). In this context, impairments in fundamental psychological capacities involved in emotion regulation, self-perception, and interpersonal functioning may be particularly relevant (Gross, 2015). To better understand the underlying mechanisms of this trauma-related coping process, recent research has focused on two interrelated constructs: structural personality organization and mentalization.

Mentalization also shares the conceptual overlap with attachment security and emotion regulation as all three constructs involve the understanding and regulation of affective experiences in interpersonal contexts (Mikulincer and Shaver, 2019). More specifically, mentalization refers to the capacity to interpret one’s own and others’ behavior in terms of underlying mental states, such as feelings, intentions, desires, and beliefs. Within the context of SUD, impairments in mentalization may, therefore, be relevant because they can contribute to difficulties in affect regulation, interpersonal functioning, and the use of substances as a coping strategy (Fonagy et al., 2018; Luyten and Fonagy, 2015). The development of mentalization ability depends on secure attachment relationships, in which the child’s inner states are appropriately reflected by their caregivers, thereby gradually developing good reflective capabilities (Fonagy and Luyten, 2009). Abuse and neglect can hinder this development and can lead to epistemic mistrust and deficits in reflective ability (Fonagy et al., 2019). Underdeveloped mentalization skills can cause emotions to be experienced as overwhelming physical states, and a lack of self-regulation skills can lead to the use of substances to regulate these affects (Bateman and Fonagy, 2016, 2019; Thorberg and Lyvers, 2010; Khantzian, 1985, 1997). While mentalization deficits are frequently observed in SUD patients (Schindler, 2019), their specific role as a mediator, aside from general structural deficits, remains to be clearly identified.

Personality organization refers to broader structural capacities of personality functioning. Contemporary psychodynamic models increasingly suggest that mentalization may be hierarchically embedded within broader personality functioning. However, both constructs were included separately in the present analyses to examine whether mentalization explains variance in addictive behavior beyond general structural personality impairment (Bateman and Fonagy, 2019; Luyten and Fonagy, 2015; Kernberg, 1984). According to Kernberg (1984), personality organization encompasses dimensions such as identity integration, defense mechanisms, and reality testing. Broader psychodynamic conceptualizations additionally include aspects related to object relations, aggression regulation, and moral functioning. Consistent with this broader framework, the Inventory of Personality Organization (IPO) additionally contains subscales addressing aggression intensity and moral values. PO is seen as a key part of personality pathology, which manifests in both the integration of self-representations and the ability to maintain stable interpersonal relationships (Caligor et al., 2018). A well-integrated personality enables individuals to smoothly adjust and better manage and regulate their affects and impulses more adaptively and to respond more flexibly to stressful situations. Traumatic relational experiences may impair the development of these structural capacities. This may be associated with uncertainties in one’s own identity, and those affected more frequently resort to primitive defense mechanisms, such as splitting or denial, to cope with stressful feelings and fears (Kernberg et al., 2008; Zimmermann et al., 2013).

Research increasingly shows that structural deficits may present one possible mechanism underlying the association between trauma and different mental health disorders. They may act as risk factors and explain how trauma is related to various mental disorders, including addictive behaviors (Freier et al., 2022; Fuchshuber et al., 2018, 2019; Insel, 2014; Kampling et al., 2022; Krakau et al., 2021). Krakau et al. (2021) showed that impairments in personality functioning significantly explained the association between maltreatment in childhood and then a person’s mental health in adulthood. It was also found that structural personality deficits led to the relationship between different types of child maltreatment and the development of depression and anxiety symptoms in a large representative sample (Freier et al., 2022). Extending this mediation perspective, it was found that both epistemic trust and personality functions acted as mediating mechanisms linking negative childhood experiences to post-traumatic stress disorder and complex post-traumatic stress disorder, underscoring the developmental importance of structural impairments following early relational trauma (Kampling et al., 2022).

Fuchshuber and Unterrainer (2020) proposed a neuropsychoanalytic framework to investigate the hypothesis that trauma-related impairments in structural PO may be related to changes in the neural systems involved in stress regulation and reward processing. Consistent with this framework, a network analysis by Fuchshuber et al. (2025a), which was used to show the interrelationships between childhood trauma and PO, identified identity diffusion and primitive defense mechanisms as central nodes within the trauma-psychopathology network. In a different study, Fuchshuber et al. (2025b) also showed that PO not only explained the association between the relationship between childhood stress and paranoid thinking but also led to it, thus highlighting its role as a vulnerability and a deciding factor in the development of psychopathology. Developmental neurobiological studies further suggest that early traumatic experiences and early-life stress may be associated with alterations in frontolimbic networks and stress-sensitive neural systems (Kirsch et al., 2020; Teicher and Samson, 2016), which have been associated with difficulties in affect regulation, impulse control, and increased vulnerability to addictive behavior (Koob and Volkow, 2016).

## 2 The current study

This study builds on the same dataset used by Renner et al. (2025), who examined the relationship between addictive behavior, primary emotions, and dissociative symptoms in the same sample. The present study expands the theoretical framework by specifically examining the role of mentalization alongside structural organization. Indirect statistical pathways involving structural personality organization and mentalization in the association between childhood trauma and addictive behavior were examined. Two studies with different methodological approaches were conducted, tailored to the respective characteristics of the samples.

In Study 1, a community sample from the general population was used to create a latent structural equation model (SEM). SEM enables the simultaneous evaluation of complex, multi-path relationships while strictly accounting for the measurement error through the use of latent variables (Kline, 2023). The present study aimed to investigate the extent to which trauma-related impairments in personality structure and mentalization ability mediate addictive behavior. To test these mediation paths, a bootstrapping procedure was used, which does not rely on the assumption that indirect paths are normally distributed (Cheung and Lau, 2008; Hayes, 2022). Personality structure was assessed using the Inventory of Personality Organization (IPO), mentalization ability using the Reflective Functioning Questionnaire (RFQ-6), and childhood trauma using the Childhood Trauma Questionnaire (CTQ). Addictive behavior was measured using the global cumulative risk score of the Alcohol, Smoking and Substance Involvement Screening Test (WHO-ASSIST). Building on these results from Study 1, Study 2 aimed to replicate and validate these associations in a sample of patients undergoing inpatient treatment for SUD (SUD sample) in an addiction treatment center in Vienna (Grüner Kreis Wien). This approach enabled the investigation of whether the psychological mechanisms identified in the community population remain stable in a sample of individuals diagnosed with SUD. The comparisons between the community and clinical samples were interpreted descriptively. Formal multigroup analyses or tests of measurement invariance were not conducted, as the primary aim of the study was not to directly compare latent structures across groups but rather to examine whether the observed associations could be descriptively replicated in a clinical SUD sample. To address the methodological challenges posed by the limited sample size (*N* = 68), a Bayesian path analysis framework was employed. This approach offers advantages, particularly with small samples, as it does not rely on the asymptotic properties of large samples (Depaoli, 2021; McNeish, 2016). In line with current recommendations for path modeling with small samples (Smid and Winter, 2020), informed priors (*μ* = 0.33, *σ* = 0.15) derived from the parameter expectations of Study 1 were included in the analysis to improve estimation stability. The inclusion of prior information in this way facilitates the precise estimation of complex signaling pathways, even under conditions of limited statistical power (van de Schoot et al., 2021). To assess the robustness of the SUD sample model, sensitivity analyses were conducted using weakly informative (*μ* = 0, *σ* = 0.5) and diffuse priors (*μ* = 0, *σ* = 10). Based on developmental and psychodynamic frameworks (Bateman and Fonagy, 2019; Kernberg, 1984), it was hypothesized that higher levels of childhood trauma in both samples would be consistently associated with greater impairments in structural personality organization and reduced mentalization abilities. The study aimed to investigate potential differences in the strength of direct and indirect pathways between the community and SUD groups.

## 3 Study 1

### 3.1 Materials and methods

#### 3.1.1 Sample and procedure

Data collection was carried out via the online platform LimeSurvey^1^ and disseminated via social media channels as well as through the University of Graz (KFU) and Sigmund Freud University Vienna (SFU). The study was based on a convenience sample, with voluntary participation and no random sampling procedure. Therefore, the findings cannot be considered statistically representative of the general population. The data were collected between December 2023 and March 2024. The inclusion criteria were legal age (≥18 years) and sufficient knowledge of German. The study was approved by the ethics committees of Karl-Franzens-University Graz (KFU) and Sigmund Freud University of Vienna (SFU). All participants provided their informed consent before the survey began. A total of 971 participants completed the questionnaire. The assessment included multiple psychodynamic and clinical self-report measures comprising approximately 108 items, including questionnaires about primary emotions (BANPS, BANPS-L), sexual feelings and behavior (Berliner Lust Skala, BYAS), experience in close relationships (ECR-RD8), gaming activity (GDT), mental stress (BSI-18), dissociative symptoms (FDS), early childhood experiences (FEE), reflective functioning (RFQ), questions about the personality organization (IPO-16), and addictive behavior (WHO-ASSIST and questions referring to nicotine use). It required approximately 60–75 min to complete the questionnaire, given the length and emotionally demanding nature of several questionnaires. In addition to insufficient knowledge of German, participant fatigue, incomplete responding, and study discontinuation may have contributed to the comparatively high proportion of missing values. After excluding cases with a high proportion of missing values, the final sample comprised 498 participants aged between 18 and 88 years (*M* = 28.01, *SD* = 11.83; Mdn = 24). A majority of the respondents identified as female (73.5%), 115 as male (23.1%), and 17 as diverse (3.4%). On average, participants had 0.17 children (*SD* = 0.54; range = 0–4). A total of 75 participants (15.1%) reported having a diagnosed mental illness, with depression (7.8%), post-traumatic stress disorder (3.6%), and anxiety disorders (3.0%) being the most common. A total of 112 participants (22.5%) reported taking medication regularly, primarily antidepressants (5.2%), hormonal contraceptives (3.6%), and thyroid hormones (2.4%). In terms of addictive behavior, 53 individuals (10.6%) reported a current or past addiction, primarily nicotine addiction (6.6% of the total sample; 62.3% of individuals who reported an addiction). Other reported addictions included behavioral addictions (1.6%), eating disorders (1.0%), and alcohol abuse (0.8%).

#### 3.1.2 Psychometric assessment

After providing their consent to the collection and processing of their personal data, the participants received a questionnaire on their sociodemographic data. This included questions about sex, age, marital status, level of education, language skills, medication, and psychiatric diagnoses.

##### 3.1.2.1 Childhood trauma

Adverse childhood experiences were measured using the Childhood Trauma Questionnaire (Bernstein and Fink, 1998, Bernstein et al., 2003; German version: Klinitzke et al., 2012). Emotional, physical, and sexual abuse, as well as emotional and physical neglect, were measured. Higher scores indicate more severe traumatic experiences. In the present sample, the CTQ showed very good internal consistency (Cronbach’s *α* = 0.90).

##### 3.1.2.2 Addictive behavior

To assess addictive behavior and substance involvement, the WHO-ASSIST was used (Humeniuk et al., 2010). The WHO-ASSIST is a screening instrument that evaluates lifetime substance use, abuse-related symptoms, and problems related to substance use, for 10 substance groups (such as “tobacco,” “alcohol,” “cannabis,” “cocaine,” “amphetamines,” “inhalants,” “sedatives,” “hallucinogens,” “opioids,” and “other drugs”). Internal consistency was high (Cronbach’s *α* = 0.94).

##### 3.1.2.3 Mentalization

Mentalization ability was measured using the German short version of the Reflective Functioning Questionnaire (Fonagy et al., 2016; German adaptation according to Spitzer et al., 2021). In accordance with the recommendations of this validation study, six of the original eight items were included (item 4, “When I get angry, I say things I later regret,” and item 7, “I always know what I’m feeling,” were excluded). The questionnaire assesses the capacity to understand one’s own and others behavior in relation to mental states, such as thoughts, feelings, and intentions. Following the scoring guidelines, higher RFQ scores reflect lower mentalization ability (hypomentalizing), whereas lower scores indicate better mentalization capacity. The scale demonstrated satisfactory internal consistency (Cronbach’s *α* = 0.81).

##### 3.1.2.4 Personality organization

Structural personality organization was evaluated using the Inventory of Personality Organization (Lenzenweger et al., 2001; German version: Zimmermann et al., 2013). The instrument measures the dimensions of identity diffusion, primitive defense mechanisms, and reality testing. Higher values indicate lower structural integration of the personality. In the present sample, the IPO showed good internal consistency (Cronbach’s *α* = 0.85).

#### 3.1.3 Statistical analysis

SEM was conducted to examine the hypothesized relationships between childhood trauma, structural personality organization, mentalization ability, and addictive behavior. All analyses were performed in *R* (version 4.4.3; R Core Team, 2025) using the *lavaan* package (version 0.6-19; Rosseel, 2012). Parameters were estimated using maximum likelihood estimator (MLR). Model fit was evaluated according to established standards (Hu and Bentler, 1999; Kline, 2023). Acceptable model fit was defined as a comparative fit index (CFI) and a Tucker–Lewis index (TLI) of ≥0.90, a root mean square error of approximation (RMSEA) of ≤0.08 with an upper limit of the 90% confidence interval of ≤0.10, and a standardized root mean square residual (SRMR) of ≤0.08. The Akaike information criterion (AIC) was used for model comparison.

**Figure 1:**
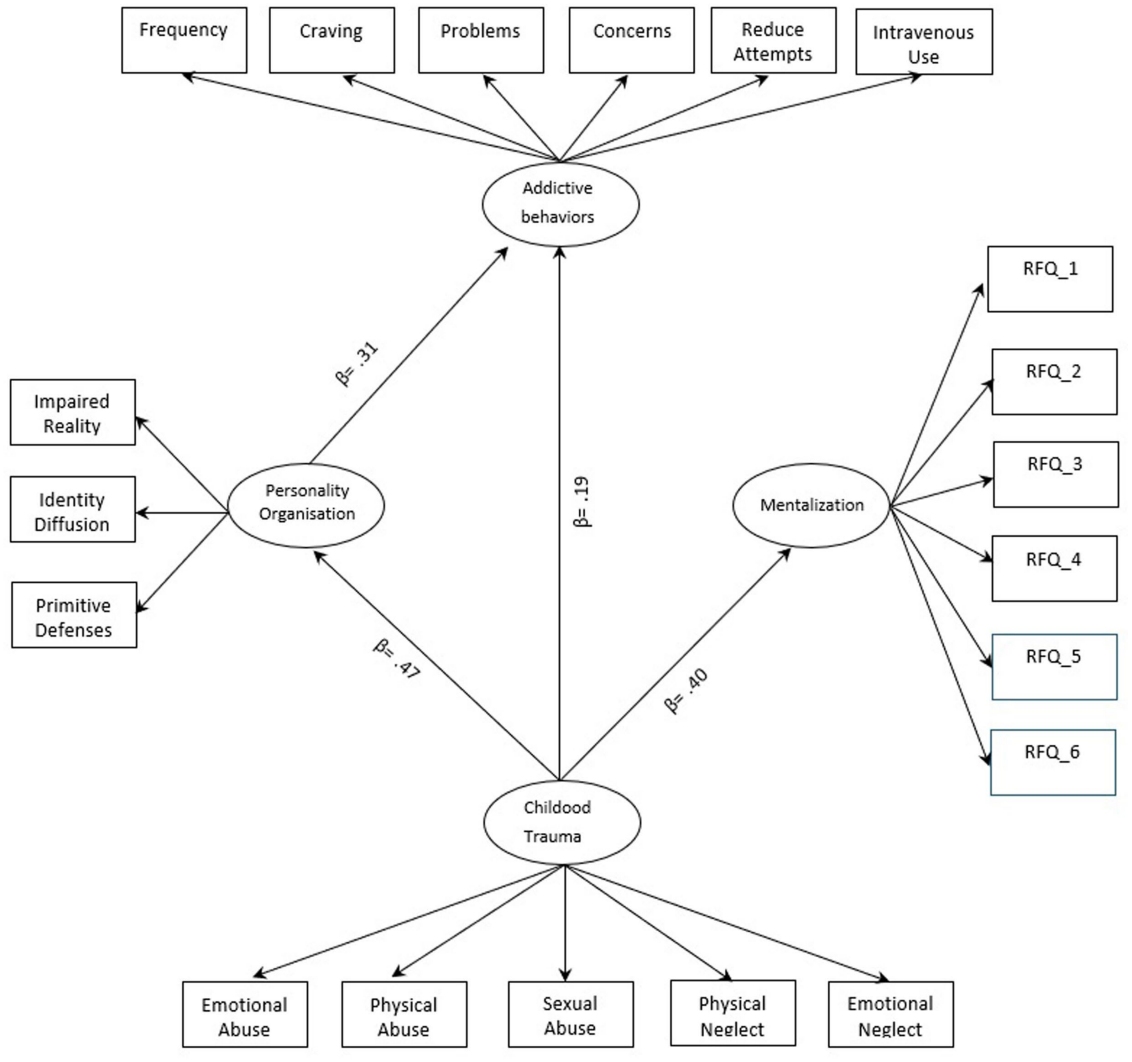
Measurement model of Study 1: childhood trauma (CTQ_lat), personality organization (IPO_lat), mentalization ability (RFQ_lat), and level of substance use (WHO_lat).

The measurement model included four latent variables (CTQ_lat, IPO_lat, RFQ_lat, and WHO_lat). Childhood trauma was represented by the five CTQ subscales: emotional abuse, physical abuse, sexual abuse, emotional neglect, and physical neglect. Personality Organization (PO) consisted of the three IPO subscales (identity diffusion, primitive defense mechanisms, and impaired reality testing). Mentalization was modeled using the six items of the RFQ-6. The items of the WHO-ASSIST were grouped into six content-defined subscales—frequency of use, craving, problems related to use (health, social, legal, or financial consequences), concern of family or friends, unsuccessful attempts to reduce use, and intravenous use. The model included direct paths from childhood trauma to personality structure, mentalization ability, and substance-related behavior, as well as indirect paths via IPO_lat and RFQ_lat. Indirect effects were estimated using bias-corrected bootstrapping (2,000 resamples; 95% confidence intervals), and effects were considered statistically significant if the confidence interval did not include zero. To ensure reproducibility, a fixed random seed was used (set.seed = 123). Psychopathological variables, such as self-reported psychiatric diagnoses, were used for descriptive characterization of the sample but were not included as covariates in the SEM as the primary aim was to test the theoretically specified pathways from childhood trauma to addictive behavior via personality organization and mentalization (Figure 1).

### 3.2 Results

#### 3.2.1 Correlations

Zero-order correlations between the variables are shown in Table 1. Childhood trauma (CTQ) showed a significant positive association with impaired personality structure (IPO; *r* = 0.41, *p* < 0.001) and reduced mentalization ability (RFQ; *r* = 0.34, *p* < 0.001). Childhood trauma was also positively associated with addictive behavior (WHO; *r* = 0.31, *p* < 0.001). Impaired personality structure demonstrated the strongest association with lower mentalization ability (*r* = 0.63, *p* < 0.001) and was moderately positively correlated with addictive behavior (*r* = 0.36, *p* < 0.001). Reduced mentalization ability demonstrated a weak but significant association with addictive behavior (*r* = 0.26, *p* < 0.001).

**Table 1:** Descriptive statistics and zero-order correlations among the Study 1 variables.

Scale	M	SD	CTQ	IPO	RFQ	WHO
CTQ	41.04	14.82	1			
IPO	29.76	8.99	0.41***	1		
RFQ	18.12	7.19	0.34***	0.63***	1	
WHO	18.62	25.42	0.31***	0.36***	0.26***	1

*N* = 498. ***p < 0.001.

#### 3.2.2 SEM results: relationships between childhood trauma, personality structure, mentalization, and addictive behavior

First, a confirmatory factor analysis (CFA) including all four latent constructs was conducted to evaluate the measurement model. The CFA demonstrated an acceptable fit to the data (CFI = 0.928, TLI = 0.917, RMSEA = 0.064, 90% CI [0.059, 0.069], and SRMR = 0.050), indicating that the latent constructs were adequately represented by their observed indicators. Subsequently, the fully hypothesized SEM, including all theoretically proposed paths, was estimated. This initial structural model showed insufficient fit (CFI = 0.891, TLI = 0.874, RMSEA = 0.079 CI [0.074, 0.087], SRMR = 0.095, and AIC = 22,251.54). Examination of the structural paths revealed that the direct effect of mentalization (RFQ_lat) on the level of substance use (WHO_lat) was not statistically significant (*β* = 0.019, SE = 0.128, *z* = 0.259, and *p* = 0.796). Following the initial estimation, this non-significant path was removed to obtain a more parsimonious model, which was then re-estimated. The respecified final model demonstrated good fit to the data (CFI = 0.928, TLI = 0.916, RMSEA = 0.068, 90% CI [0.061, 0.074], SRMR = 0.050, and AIC = 22,644.481). Although the AIC increased slightly compared to the initial model, the respecified model was retained due to improved global fit indices and greater theoretical interpretability (Table 2). The final model supported the assumption that childhood trauma was associated with higher structural personality impairment, which, in turn, was related to increased addictive behavior. Mentalization did not show a direct association with addictive behavior. In the measurement model, the four latent constructs (CTQ_lat, IPO_lat, RFQ_lat, and WHO_lat) were specified with freely estimated covariances. This reflects the assumption that childhood trauma, structural personality functions, mentalization ability, and addictive behavior are interrelated but distinct domains. No causal relationships are implied at this level of measurement (see Tables 3, 4).

**Table 2:** Model fit indices for the hypothesized and nested structural equation models.

Model	CFI	TLI	RMSEA	90% CI RMSEA	SRMR	AIC
Hypothesized model	0.891	0.874	0.079	[0.074, 0.087]	0.095	22,251.54
Nested model	0.928	0.916	0.068	[0.061, 0.074]	0.050	22,644.481

The nested model excluded the non-significant structural path from mentalization ability (RFQ_lat) to substance-related risk behavior (WHO_lat); CFI = comparative fit index; TLI = Tucker–Lewis index; RMSEA = root mean square error of approximation; SRMR = standardized root mean square residual.

**Table 3:** Standardized factor loadings of the measurement model.

Scale	Subscale	*λ*
CTQ	Emotional abuse	0.85
	Physical abuse	0.56
	Sexual abuse	0.45
	Emotional neglect	0.78
	Physical neglect	0.62
IPO	Identity diffusion	0.81
	Primitive defenses	0.85
	Reality testing	0.79
RFQ	RFQ1	0.45
	RFQ2	0.70
	RFQ3	0.62
	RFQ4	0.69
	RFQ5	0.84
	RFQ6	0.56
WHO	Frequency	0.79
	Craving	0.85
	Problems related to use	0.81
	Expressed concerns	0.83
	Attempts to reduce use	0.84
	Intravenous use	0.27

**Table 4:** Direct, indirect, and total effects from the structural equation model.

Effect	Path	*β*	*p*	95% CI
Direct effects	CTQ_lat → IPO_lat	0.47	<0.001	[0.37, 0.57]
	CTQ_lat → RFQ_lat	0.40	<0.001	[0.29, 0.50]
	IPO_lat → WHO_lat	0.31	<0.001	[0.20, 0.41]
	RFQ_lat → WHO_lat	–	>0.05	–
	CTQ_lat → WHO_lat	0.19	0.002	[0.07, 0.30]
Indirect effects	CTQ_lat → IPO_lat → WHO_lat	0.14	<0.001	[0.09, 0.20]
Total effect	CTQ_lat → WHO_lat	0.33	<0.001	[0.23, 0.43]

#### 3.2.3 Direct effects

The analysis demonstrated that childhood trauma was significantly associated with greater structural impairment of personality and reduced mentalization ability. Higher levels of childhood trauma predicted greater structural impairment (*β* = 0.47, 95% CI [0.37, 0.57], *p* < 0.001) and reduced mentalization ability (*β* = 0.40, 95% CI [0.29, 0.50], *p* < 0.001). Structural personality impairment was significantly positively associated with addictive behavior (*β* = 0.31, 95% CI [0.20, 0.41], *p* < 0.001). The direct effect of childhood trauma on addictive behavior was also significant (*β* = 0.19, 95% CI [0.07, 0.30], *p* = 0.002).

#### 3.2.4 Indirect effects

The results showed a significant indirect effect of childhood trauma on addictive behavior via structural personality impairment (*β*_ind_ = 0.14, 95% CI [0.09, 0.20], *p* < 0.001). The direct effect of childhood trauma on addictive behavior remained significant. These results indicate a partial mediation. The total effect of childhood trauma on addictive behavior combining direct and indirect pathways was also significant (*β*total = 0.33, 95% CI [0.23, 0.43], *p* < 0.001).

## 4 Study 2

### 4.1 Materials and methods

#### 4.1.1 Sample and procedure

The data for the SUD sample were collected during the same time period as those in Study 1. For this study, paper questionnaires were distributed to 75 patients at the Grüner Kreis organization. All participants were undergoing inpatient treatment for a diagnosed SUD. Seven individuals were excluded due to missing data. The questionnaires were exactly the same as those used in Study 1. The final SUD sample consisted of 68 participants aged between 19 and 72 years (*M* = 35.96, *SD* = 12.62). A majority of the participants were male (76.5%), while 23.5% were identified as female. In addition, a majority of the participants were single (70.6%); a small number reported being in a relationship (5.9%), married (10.3%), divorced (11.8%), or widowed (1.5%). Nearly half of the sample had completed professional or technical training (48.5%), followed by a compulsory school diploma (27.9%) and a high school diploma (19.1%). Only a small proportion of participants reported having an academic degree (≤1.5%). With regard to substance-related disorders, alcohol-related problems were reported most frequently (36.8%), followed by the use of stimulants (25.0%), opioids (17.6%), and benzodiazepines (11.8%). Polydrug use was reported by 13.2% of the sample. The inclusion criteria were a minimum age of 18 years and current or previous psychiatric or psychotherapeutic treatment.

### 4.2 Statistical analysis

The analyses were performed in *R* (version 4.4.3; R Core Team, 2025) using the *blavaan* package (Merkle and Rosseel, 2018), which extends the *lavaan* framework for Bayesian structural equation modeling (Gabry et al., 2019; Rosseel, 2012). Model fit was evaluated using the posterior predictive *p*-value (PPP), where values of approximately 0.50 indicate an optimal fit between the observed data and the model-implied data (Gelman et al., 2013; Smid and Winter, 2020). A latent Bayesian SEM was specified equivalent to Study 1. A preliminary evaluation of the measurement model indicated weak and inconsistent factor loadings for the latent substance use construct (WHO_lat). Specifically, items reflecting active consumption behavior (e.g., intravenous use and consumption frequency) showed weak correlations with subjective items (e.g., craving and health problems), likely due to the abstinence requirement in the inpatient setting. Combined with the small sample size, this led to model convergence issues and poor fit (PPP = 0.001). Consequently, the analysis was shifted to a Bayesian path model using manifest variables to ensure robust estimation. The initial model including all parameters (CTQ, IPO, RFQ, and WHO) still showed a weak fit (PPP = 0.313). After removing the RFQ–WHO path, the fit improved only slightly (PPP = 0.321). When RFQ was excluded from the model entirely, the fit increased further (PPP = 0.480). The final model therefore retained CTQ, IPO, and WHO. To examine the robustness of these results and account for the limited data availability, a sensitivity analysis was performed comparing three different prior specifications. These included a model with default diffuse priors [*N*(0, 10)], a model with weakly informative priors [*N*(0, 0.5)], and a final model utilizing informed priors [*N*(0.33, 0.15)] derived from the empirical findings of Study 1. While all three path specifications demonstrated excellent global fit (PPP ranging from 0.480 to 0.519), the models relying on diffuse and weakly informative priors yielded wide credibility intervals that consistently crossed zero. The Bayesian path model with informed priors demonstrated a good overall fit to the data (PPP = 0.480).

## 5 Results

### 5.1 Direct effects

Using the informed priors, childhood trauma was significantly associated with structural personality impairment. The posterior mean for the unstandardized effect was *B* = 0.30 (PSD = 0.11, 95% CI [0.10, 0.51]), corresponding to a standardized effect of *β* = 0.24. Similarly, structural personality impairment significantly predicted addictive behavior of *B* = 0.23 (PSD = 0.10, 95% CI [0.04, 0.43], *β* = 0.22). In contrast to Study 1, the direct path from childhood trauma to addictive behavior was not significant, with the 95% credibility interval crossing zero with *B* = 0.17 (PSD = 0.11, 95% CI [−0.05, 0.39], *β* = 0.12).

### 5.2 Indirect effects

The analysis of the indirect effect showed a posterior mean of *B*_ind_ = 0.07 (PSD = 0.04). However, the 95% credibility interval ranged from −0.01 to 0.15, slightly including the value zero. While the direction of the effect is consistent with Study 1 (*β*_ind_ = 0.05), it does not reach strict statistical significance in the smaller SUD sample. The total effect was positive, and its credibility interval excluded zero (*B*_total_ = 0.24, 95% CI [0.01, 0.46], *β*_*t*otal_ = 0.17), suggesting an overall association between trauma and addictive behavior in the SUD group (see also Table 5).

**Table 5:** Bayesian path model with informed priors (SUD sample, *N* = 68).

Effect	Path	*B*	Post. SD	95% CI	*β*
Direct effects	CTQ → IPO	0.30	0.11	[0.10, 0.51]	0.24
	IPO → WHO	0.23	0.10	[0.04, 0.43]	0.22
	CTQ → WHO	0.17	0.11	[−0.05, 0.39]	0.12
Indirect effect	CTQ → IPO → WHO	0.07	0.04	[−0.01, 0.15]	0.05
Total effect	CTQ → WHO	0.24	–	[0.01, 0.46]	0.17

## 6 Discussion

The primary aim of this study was to identify psychological mechanisms linking childhood trauma to addictive behavior, with the focus on the mediating role of PO and mentalization. The results from both studies can be interpreted within the framework of developmental and psychodynamic theories. From this perspective, early adverse childhood experiences have been associated with impairments in self-regulation skills and increased vulnerability to psychopathology and addictive behavior (Felitti et al., 1998; Khantzian, 1985; Bateman and Fonagy, 2019). In both samples, childhood trauma was significantly associated with impairments in structural personality organization, which is also consistent with Kernberg’s (1984) object relations model that suggests that early relational trauma can impair the integration of positive and negative experiences with attachment figures and can lead to long-term structural impairments. This interpretation may also be in line with neurobiological findings. Trauma during sensitive developmental stages may be associated with changes in brain regions involved in affect regulation and impulse control, given that impaired impulse control has repeatedly been associated with an increased vulnerability for substance use disorders (Kirsch et al., 2020; Koob and Volkow, 2016; Teicher and Samson, 2016).

In Study 1, the mediation path was statistically significant, with structural impairments partially explaining the association between childhood trauma and addictive behavior, which is consistent with the assumption that personality structure may serve as a regulatory factor in the general population (Freier et al., 2022; Krakau et al., 2021). Lower levels of structural integration were associated with higher addictive behavior. Difficulties in structural personality integration may be associated with reduced affect regulation capacities and a greater tendency to use substances as a maladaptive coping strategy. This perspective is reflected in the self-medication hypothesis (Khantzian, 1997, 2013; Suh et al., 2008), which proposes that substance use following early traumatic experiences may, in part, serve as a means of regulating underlying structural impairments (Costanzo et al., 2023; Hawn et al., 2020).

In Study 2, childhood trauma was statistically associated with impairments in structural personality organization. These, in turn, were related to addictive behavior. From this perspective, substance misuse may be understood as one of the several possible expressions of impaired self-regulatory integration (Caligor et al., 2018; Levy et al., 2006). The absence of a direct association between childhood trauma and addictive behavior in Study 2 should be considered in the context of the study conditions. In contrast to Study 1, in which natural variations in substance use were observed, the SUD sample was assessed within a controlled inpatient setting focused on abstinence. Therefore, the WHO-ASSIST scores in Study 2 may reflect past patterns of substance misuse and subjective craving rather than acute addictive behavior. Given that participants were assessed within a structured inpatient rehabilitation setting characterized by abstinence requirements, restricted access to substances, and ongoing therapeutic support, variability in current substance use was likely reduced. Accordingly, the observed associations in the clinical sample may have been more strongly related to enduring psychological burden and structural vulnerabilities than to current substance use behavior itself.

This contextual factor may have weakened the observable direct relationship between trauma and addictive behavior within the statistical model. In Study 2, the direct association between childhood trauma and addictive behavior was not statistically significant. Similarly, the specific indirect pathway via structural personality organization did not achieve statistical significance although the credibility interval only slightly exceeded zero. It is important to point out that the overall effect of childhood trauma on addictive behavior remained statistically credible, suggesting a robust overall association. This difference compared to the community sample is likely due to methodological and sampling-related characteristics. The current findings suggest that, within the present model, structural personality impairment emerged as the strongest and most consistent correlate of addictive behavior in the clinical sample. This may indicate that, in individuals with more severe clinical presentations, substance-related burden is closely associated with broader impairments in personality functioning. In this scenario, the influence of childhood trauma may be reflected in current structural impairments, which were more strongly associated with addictive behavior in the present model. Studies of individuals with severe personality pathology and high trauma exposure have shown that structural personality impairments occur frequently. These impairments are associated with far-reaching difficulties in regulating cognition, emotions, and behavior (Sharp et al., 2011; Wright and Simms, 2016). This concept is also referred to in current literature as the “p-factor,” which suggests that severe structural impairments function as a central vulnerability for general psychopathology (Caspi et al., 2024). This is consistent with the recent paradigm shift in the ICD-11 and the “Alternative Model for Personality Disorders” in the DSM-5. According to this model, the general level of personality functioning is viewed as the transdiagnostic core of personality pathology (Bach and First, 2018; Hopwood et al., 2018; Zimmermann et al., 2019). In samples of individuals with severe general functional impairments, who often have multiple mental disorders and comorbid conditions, more specific associations, such as the association between trauma and addictive behavior, may be less clearly visible in statistical models (Caspi et al., 2024).

In line with theories of epistemic mistrust (Fonagy et al., 2019; Fonagy et al., 2016; Kampling et al., 2022), Study 1 showed that a greater extent of childhood trauma is associated with lower mentalization ability. Explanatory models suggest that early trauma may impair trust in others and the capacity for self-reflection (Jurist, 2005). Although mentalization deficits were associated with childhood trauma, they did not show an independent association with addictive behavior once structural personality organization was considered in the model. This suggests that their statistical contribution could be partly explained by more comprehensive structural impairments. This lack of an independent statistical mediation pathway should not be seen as an indication of the irrelevance of the concept. Instead, it points to conceptual overlaps between mentalization and general personality structure (Zettl et al., 2020). From this perspective, the findings may support the assumption that mentalization represents a more specific psychological capacity operating within broader domains of personality functioning rather than a fully independent mechanism. Both instruments partly assess capacities related to self-reflection, affect regulation, and interpersonal functioning, which may have reduced the ability to detect unique effects of mentalization within the structural models (Marszał and Górska, 2015). It is possible that, in a unified model, the broader transdiagnostic construct of structural personality impairment takes precedence, causing the significance of mentalization ability to recede into the background (Luyten et al., 2020). Additionally, measurement constraints of the RFQ-6 short form may have contributed to the absence of distinct effects as the brief format may not sufficiently capture fluctuations between hypermentalizing and hypomentalizing processes frequently observed in addictive disorders (Choi-Kain and Gunderson, 2008; Taubner and Curth, 2013; Taubner and Volkert, 2016).

In the SUD sample, structural personality impairment emerged as the most consistent correlate of addictive behavior within the model. This is supported by other clinical studies, which show that hospitalized patients with personality impairment are more likely to engage in impulsive and addictive behaviors (Gratz and Tull, 2010; Weiss et al., 2012). These findings are compatible with theoretical and psychodynamic treatment models that emphasize personality structure as a central dimension in the treatment of severe psychopathology (Kernberg et al., 2008; Yeomans et al., 2015). From this perspective, addiction among individuals who have experienced trauma in the past can be understood not merely as a behavioral problem but as part of broader structural vulnerabilities. Interventions focusing on identity integration and the development of more adaptive coping mechanisms could therefore represent a transdiagnostic approach aimed at strengthening general self-regulation abilities (Unterrainer, 2025; Clarkin et al., 1999).

The findings of the present study are consistent with theoretical perspectives suggesting that the addictive behavior in the context of traumatic childhood experiences may, in part, reflect attempts to maintain emotional and psychological stability (Costanzo et al., 2023; Khantzian, 1997). From this perspective, abstinence alone may not entirely address the underlying structural vulnerabilities associated with SUD. Additional corrective emotional experiences and a therapeutic relationship are important and can serve as a stable framework for building trust and strengthening the capacity for self-reflection (Bateman and Fonagy, 2019; Yeomans et al., 2015). In this context, the present findings could support treatment approaches that focus not only on symptom reduction but also on the long-term development of psychological structures (Unterrainer, 2025).

## 7 Limitations and future perspectives

Despite a careful methodological approach, it is important to mention the limitations of this study. Although statistical mediation models were tested, the present analyses are based on cross-sectional data and the identified indirect pathways should therefore be interpreted as statistical associations rather than evidence of temporal or causal processes. Alternative causal relationships between the study variables cannot be ruled out. For example, addictive behavior itself may contribute to impairments in personality functioning or reflective capacity, and reciprocal associations between these constructs are also theoretically plausible (Kline, 2023). Although several model respecifications and sensitivity analyses were conducted, these primarily addressed statistical parsimony and the robustness of Bayesian estimates rather than substantively different theoretical models. Therefore, competing pathways, including reversed or reciprocal associations and alternative mediation structures, were not formally tested. Given the cross-sectional design, the directionality of these associations cannot be determined. Future longitudinal studies are needed to compare competing theoretical models and clarify causal relationships among childhood trauma, personality functioning, mentalization, and addictive behavior (Bateman and Fonagy, 2019; Schindler, 2019).

Regarding the data collection performed in Study 1, the recruitment via social media and university-related platforms may have introduced self-selection biases as individuals with greater interest in psychology, higher educational levels, or increased willingness to reflect on personal experiences may have been more likely to participate. Furthermore, the comparatively high exclusion rate may have affected the representativeness of the final sample. This may partly be attributable to the relatively long assessment procedure, which consisted of 108 questionnaire items and may have increased the likelihood of incomplete responses or study discontinuation. It is possible that individuals with higher symptom burden or lower concentration were somewhat less likely to complete the assessment procedure, potentially resulting in a final sample with slightly higher functioning. Relatedly, the present models did not account for potential confounding variables. Although psychiatric diagnoses and symptom-related variables, such as depression, anxiety, and post-traumatic stress symptoms, were available descriptively, these variables were not included in the structural analyses. Socioeconomic status and other clinically relevant factors were also not considered. Therefore, the observed associations between childhood trauma, personality organization, mentalization, and addictive behavior should be interpreted within the boundaries of the selected theoretical model. Future studies should include additional clinically relevant variables as covariates, such as depressive symptoms, post-traumatic stress symptoms, and socioeconomic status, to assess the robustness of the observed associations. Another restriction applies to the assessment of addictive behavior in the clinical sample. Given that the participants were undergoing inpatient treatment to achieve abstinence, their substance use was limited at the time of the survey. The WHO-ASSIST scores in this group therefore likely reflect the subjective burden of addiction, such as craving or past substance-related difficulties, rather than current use. Due to the inpatient setting, the participants likely differed less in their actual substance use than they would under naturalistic conditions. This restriction of variability may have weakened the observable association between childhood trauma and current addictive behavior within the statistical models. In naturalistic settings, traumatic experiences may be more directly associated with maladaptive coping through active substance misuse, particularly during periods of emotional distress or impaired self-regulation. However, within the structured inpatient environment, acute consumption behavior was externally restricted through abstinence requirements. Therefore, trauma-related differences may have been expressed less through current addictive behavior itself and more through persistent structural impairments, emotional dysregulation, or subjective craving. Additionally, childhood trauma was assessed retrospectively using self-report measures, which may be influenced by recall bias, current emotional state, or subjective reinterpretation of past experiences. Although the CTQ is a well-established instrument, retrospective assessments of childhood adversity may not fully correspond to objectively verifiable experiences (Bernstein et al., 2003).

All collected data were based on self-reports. These primarily reflect conscious assessments, while unconscious processes, particularly those related to self-regulation and emotional regulation, are only recorded to a limited extent (Panksepp and Biven, 2012). In this context, the use of self-reports poses a particular methodological challenge, as individuals with severe structural impairments may show reduced reflective capacities, potentially limiting the accuracy of self-report assessments of mentalization ability. For future studies, it might be beneficial to rely more heavily on methods based on expert-rated clinical interviews (e.g., structured interviews on personality functioning) in addition to self-report measures. In this study, both personality organization and mentalization ability can be assessed in a more nuanced and less biased manner. In particular, interview-based measures of reflective functioning or methods such as “Movie for the Assessment of Social Cognition” (Dziobek et al., 2006) may provide a more ecologically valid assessment of mentalization processes, especially in clinical populations characterized by impaired self-awareness, affect dysregulation, or unstable self-representations (Zettl et al., 2020).

In addition, the WHO-ASSIST combines multiple dimensions of substance involvement, including frequency of use, craving, social consequences, and previous attempts to reduce consumption, into a global cumulative risk score. While this allows for a broad assessment of addictive behavior, it may obscure potentially meaningful differences between substances and between current versus past substance-related difficulties. Particularly in the inpatient SUD sample, some indicators may have reflected retrospective burden or subjective craving rather than ongoing substance use behavior, which may have reduced the specificity of the construct within the statistical models. The comparatively weak factor loading of the intravenous use indicator in Study 1 likely reflects the characteristics of the community sample, in which intravenous substance use was reported only rarely. As a result, this indicator may have contributed less strongly to the overall latent substance use construct compared to broader indicators, such as craving or substance-related problems. Furthermore, the WHO-ASSIST assesses 10 different substance groups. Substances such as nicotine or alcohol can be linked with different psychological mechanisms. Therefore, future research could investigate whether different psychopathological patterns are associated with specific types of addictive behavior (Han et al., 2021; Koob and Volkow, 2016; Verdejo-García et al., 2008). If the individual classes of substances are considered separately, specific relationships, such as those with PO or mentalization, might become clearer and more distinct.

Although Bayesian analysis using informed prior distributions helped stabilize the estimates despite the sample size, the relatively small clinical sample may also have reduced statistical stability and limited the generalizability of the observed associations. In addition, the heterogeneous composition of the SUD sample and the comparatively low proportion of female participants could limit the generalizability of the results. The markedly different gender distributions across the two samples, with a higher proportion of men in the SUD sample and a higher proportion of women in the community sample, may limit the comparability and generalizability of the findings, particularly given known gender differences in trauma exposure, emotional processing, and addictive behavior (e.g., Belfrage et al., 2023; Langeland and Olff, 2024). Furthermore, the inpatient clinical sample consisted exclusively of treatment-seeking individuals undergoing abstinence-oriented rehabilitation. The findings may therefore not generalize to individuals with SUD in outpatient settings, active substance use phases, or non-treatment populations. In addition, both samples were recruited exclusively from Austria living populations, which may limit the cross-cultural generalizability of the results to healthcare systems, sociocultural environments, or substance use patterns in other regions. The findings highlight the importance of larger and more diagnostically differentiated samples to better understand the complex relationships between trauma, structural personality organization, mentalization, and the development of addiction (Hughes et al., 2017; Vonderlin et al., 2018).

## 8 Conclusion

The present findings emphasize the relevance of early traumatic experiences in the context of addictive. In the community sample, structural personality impairment explained part of the association between childhood trauma and addictive behavior. However, this statistical mediation pattern was not fully replicated in the SUD sample. Although patients reported higher levels of trauma exposure and structural impairments, the association with current substance use was less pronounced. This could be partly due to the influence of the inpatient treatment environment and an overall higher level of psychopathological distress. The results suggest that early traumatic experiences are closely associated with structural aspects of personality functioning. Therefore, interventions should focus on structural integration, emotional regulation, and reflective capacity in addition to symptom relief.

## Data Availability

The raw data supporting the conclusions of this article will be made available by the authors, without undue reservation.

## Footnotes

1
https://www.limesurvey.org/
